# The Influence of Health Behaviours in Childhood on Attention Deficit and Hyperactivity Disorder in Adolescence

**DOI:** 10.3390/nu8120788

**Published:** 2016-12-02

**Authors:** Xiuyun Wu, Arto Ohinmaa, Paul J. Veugelers

**Affiliations:** School of Public Health, University of Alberta, 350 University Terrace, 8303—112 Street, Edmonton, AB T6G 2T4, Canada; xiuyun@ualberta.ca (X.W.); paul.veugelers@ualberta.ca (P.J.V.)

**Keywords:** children, adolescents, attention deficit and hyperactivity disorder, diet quality, physical activity, sedentary behaviour

## Abstract

Attention-deficit and hyperactivity disorder (ADHD) in children and adolescents is a global public health burden. Identification of health-related behavioral risk factors including diet quality and physical and sedentary activities for ADHD is important for prioritizing behavioral intervention strategies to improve mental health. This study aimed to examine the association of diet quality, physical activity, and sedentary behaviours in childhood with ADHD throughout adolescence. We linked data from grade five students aged primarily 10 and 11 years old who participated in a population-based lifestyle survey in the Canadian province of Nova Scotia with their administrative health care data. We applied negative binomial regression methods to examine the associations between health behaviours and ADHD. Of the 4875 students, 9.7% had one or more diagnoses of ADHD between the ages of 10/11 and 18 years. The number of primary diagnoses with ADHD was statistically significantly lower among students with better diet quality, higher levels of physical activity, and those that spent less time playing computers and video games (*p* < 0.05). These findings suggest that health promotion programs aiming to improve children’s diets and active lifestyles may also reduce the public health burden of ADHD.

## 1. Introduction

Attention-deficit and hyperactivity disorder (ADHD) is one of the most frequently diagnosed mental health disorders among children and adolescents, and has been acknowledged as a global public health burden [1]. Epidemiological studies and meta-analyses have reported that about 5% to 12% of school age children and adolescents experience ADHD, with a higher prevalence among boys than girls [2,3,4,5]. ADHD is a neurobehavioral disorder, characterized by manifestations of inattention, hyperactivity, and impulsivity [6]. ADHD in childhood and adolescence often persists into adulthood, causing significant impairment in psychosocial, neurobehavioral, and cognitive functioning [7]. Children and adolescents with ADHD are more likely to experience difficulties in academic learning and school performance [8,9,10,11], and have adverse behaviours and health consequences, including bullying behaviour, suicidal ideation, poor psychosocial health, increased injuries [12,13,14], and low quality of life [15,16]. ADHD commonly co-occurs with other mental health disorders, such as conduct disorder and internalizing disorder, which may exacerbate the difficulties of affected individuals [17]. Children and adolescents with ADHD utilize excessive health care services that incur substantial health care costs [18].

Studies in the etiology of ADHD have predominantly focused on genetic and environmental factors [19,20]. Some studies have investigated socio-demographic factors for ADHD, such as gender, age, socio-economic status, and overweight and obesity [21,22,23]. However, very few have examined lifestyle behaviours including diet quality, and physical and sedentary activities among children and youth. Several cross-sectional and case-control studies have reported that the unhealthy dietary patterns are related to an increased risk of ADHD [24,25,26,27]. An unhealthy dietary pattern, characterized as a diet high in sugar, salt, and saturated and total fat, while low in whole grains, fish, fruits, and vegetables, was associated with increased ADHD symptoms [24,27]. Children and youth with poor diets are likely to consume less vitamins, minerals, and fatty acids such as omega-3 and omega-6, that have been suggested to be involved in the development of ADHD [25]. We are not aware of any longitudinal studies of the impact of overall diet quality on ADHD in children and adolescents whereas such studies provide stronger evidence than the above discussed cross-sectional and case-control studies.

Cross-sectional and systematic review studies that examined the associations between physical activity (PA) and ADHD provide some support for the positive effects of PA on alleviating inattentive and hyperactive symptoms of ADHD [21,28,29,30]. Very few prospective studies have tested the effect of PA on ADHD in adolescence [31], especially in population-based studies. Research findings for the association between ADHD and sedentary behaviours characterized by spending increased amount of time on the use of computer/video games and watching television (TV) have been inconsistent. Some reported a negative effect of sedentary behaviours on attention problems and/or ADHD [32,33,34], while others did not show a significant relationship between them [35,36]. Moreover, in the previous studies the identification of individuals with ADHD was mainly based on parent- or teacher-reporting or youth self-reporting [32,33,34,35,36]. None of these studies utilized physician diagnosis of ADHD, which is more accurate than questionnaire based methods.

Further investigation of the predictive effect of these health-related behaviours on ADHD will help elucidate prospective relationships between diet quality, specific types of PAs and sedentary behaviours, and the future development of ADHD. The information is important for public health policy makers to allocate scarce resources to the health interventions and preventive actions targeted at promoting healthy diets and active living behaviours among youth to reduce the burden from ADHD and to enhance their mental health.

The objective of the present study is to investigate the association between diet quality, physical activity and sedentary behaviours, and physician diagnosed ADHD throughout adolescence in a cohort of grade five students.

## 2. Methods

### 2.1. The Survey

The 2003 Children’s Lifestyle and School Performance Study (CLASS) is a population-based survey among grade five students who are primarily 10 and 11 years old, and their parents, in the province of Nova Scotia, Canada. Of all 291 provincial public schools with grade five students, 282 schools participated in the study. The participation rate was 51.1% per school. Of the 5517 grade five students in the participating schools who received parental consent to participate in the study, 5200 students (94.3%) completed the surveys [37].

The CLASS study consisted of a home survey that was completed by parents, a student survey that included questions on the frequency of physical activities, self-esteem, and time of sedentary behaviours, and a Canadian version of the Harvard Youth/Adolescent Food Frequency Questionnaire (YAQ) [38]. The YAQ measure has been previously validated and extensively used in this population of children [39]. Student surveys were administered in the schools by study assistants who also measured standing height of the students to the nearest 0.1 cm and body weight to the nearest 0.1 kg on calibrated digital scales. The home survey collected information on the children’s place of residency, gender, household income, and highest level of parental education. In addition, parents provided the Nova Scotia health insurance number for their child and consented to allow this to be used for future data linkage with the administrative health databases.

### 2.2. Administrative Health Data

The administrative health data were derived from the Medical Services Insurance (MSI) database and the Canadian Institute for Health Information Discharge Abstract Database (CIHI DAD). The MSI database is administered by Medavie Blue Cross for the province of Nova Scotia and contains records for each insured health service rendered by a physician (including emergency room visits) and paid for by the Nova Scotia provincial health care system. The CIHI DAD contains a comprehensive administrative transcription of each admission to a Nova Scotia hospital facility. Both of these databases contain individual patient-level information including patient demographic characteristics, visiting physicians, and diagnoses received. Data were available from 1992 (when the grade 5 students who participated in 2003 were born) to 2011 (when participating students turned 18 years of age).

Of the 5200 students who completed the survey, 4875 (94%) were provided valid health card numbers and were successfully linked with the administrative health data.

### 2.3. Assessment of Health Behaviours

The exposure variables in the present study were diet quality, PA and sedentary behaviours among grade five students. On the basis of students’ nutrient intake and dietary information from the YAQ [38] and Canadian Nutrient Files, we calculated the intake of nutrients and energy as well as the number of daily servings of fruits and vegetables. We then calculated the diet quality index international (DQI-I) [40]. The DQI-I scores range between 0 and 100, with higher scores indicating a better diet quality. The DQI-I constitutes four components: variety, adequacy, moderation, and overall balance of the diet. We divided the DQI-I scores into tertiles with a higher tertile indicating better diet quality.

The CLASS included questions from the National Longitudinal Survey for Children and Youth [41] on playing sports or physical activities with and without a coach. The questions were categorized as weekly times of engagements in the physical activities: Never, 1 to 3 times/week, and ≥4 times/week. The questions for sedentary behaviours were reported as daily hours spent on playing computer or video games, and on watching television: less than 1 h/day, 1–2 h/day, 3–4 h/day, and ≥5 h/day as response categories.

### 2.4. The Outcome of Attention Deficit and Hyperactivity Disorder

Participants are considered to have an attention deficit and hyperactivity disorder if they received one or more diagnoses of attention deficit disorder, hyperactivity disorder, hyperkinetic syndrome, or hyperkinetic conduct disorder according to the International Classification of Diseases, Ninth Revision, Clinical Modification (ICD-9-CM) or Tenth Revision, (ICD-10-CA). The ICD-9 and ICD-10 codes for ADHDs in this study include 314.xx (ICD-9-CM) and F90.xx (ICD-10-CA). All the primary diagnoses for ADHDs between 2003 (children’s age 10 to 11 years) and 2011 (participants turned 18 years of age) were considered.

### 2.5. Confounders

We considered gender, household income, parental educational attainment, residential location, body weight status, and self-esteem as potential confounders in the relationship between the health behaviours and ADHD. Each of these variables are established predictors for ADHD [21,23,42]. The household income was grouped into four levels: $0 to $20,000; $20,001–$40,000; $40,001–$60,000; >$60,000. Parental educational attainment was entered as secondary school or less; college; and university or above. Residential location was classified as urban and rural. We applied the age- and gender-specific body mass index cut-off points for children that were established by the International Obesity Task Force [43]. Body weights was categorized as normal weight, overweight, and obesity.

Eleven questions on the measurement of self-esteem of the students were included in the CLASS survey, each with three response levels: (1) Never or almost Never; (2) Sometimes and (3) Often or almost Always [44]. By means of principal component analysis (PCA), we reduced the questions to four components: self-perception, externalizing problems, internalizing problems, and social-perception [45]. The estimated self-esteem scores for each of these four components were dichotomized using the quantiles with an equal size of the two groups. The binary variable for self-esteem was included in the regression analysis as self-esteem is strongly associated with mental health [42,45].

### 2.6. Statistical Analysis

We applied univariate (unadjusted) and multivariable (adjusted) negative binomial regression models (NBMs) to examine the association between diet quality, PA, sedentary behavior, and the diagnosis of ADHD. The multivariable NBM was adjusted for the confounding influence of students’ gender, residency, household income, parental education, body weight, and self-esteem. The multivariable regression was also adjusted for energy intake as per established recommendations for the analysis of food frequency data [46]. The number of health care provider contacts with a diagnosis of an ADHD is over-dispersed, where the variance (=12.45) was greater than its mean (=0.79), thus the NBM was an appropriate method to analyze counts [47]. We conducted gender stratified analyses to examine whether the effect of the health behaviours on the health care provider contacts for ADHD is different for boys and girls.

As response rates in residential areas with lower household incomes were slightly lower than the average, we weighted the analyses using response weights such that the estimates represent the population of grade five students in the province of Nova Scotia [37]. Missing values for household income, parental education, and body weight status were considered as separate covariate categories in the regression models [48]; the estimates were not presented. The statistical analysis was conducted using the STATA 13 statistical software package (Stata Corp., College Station, TX, USA).

### 2.7. Research Ethics

The data collection and parental informed consent forms of the CLASS study were approved by the Human Research Ethics Boards of Dalhousie University and the University of Alberta. The data linkage of the CLASS survey data with the administrative health data was approved by the Human Research Ethics Boards of the University of Alberta and Dalhousie University, and by Health Data Nova Scotia, the custodian of the administrative health data.

## 3. Results

Of the 4875 students, 50.8% were girls. 9.7% (*n* = 469) had one or more health care provider contacts with a diagnosis of ADHD following the 2003 CLASS survey. Students who reported a poorer diet quality, reported less PA, and reported more time using a computer or playing video games in the 2003 survey, were more likely to be diagnosed with ADHD in subsequent years (Table 1). A diagnosis of ADHD was also more prevalent among students from families with low income and less parental education, and among students who live in urban areas and reported low self-esteem (Table 1). Boys received more diagnoses (14.5%) for ADHD than girls (5.1%). Obese children were less likely to be diagnosed with ADHD relative to their normal weight peers.

Table 2 shows the incidence rate ratio (IRR) and 95% confidence intervals (CIs) for the number of diagnoses of ADHD associated with the health behaviours after adjusting for the background variables. Children in the highest tertile for diet quality had 0.49 times lower number of health care provider contacts for ADHD (IRR = 0.51, 95% CI: 0.35, 0.75) relative to children in the lowest tertile. There was not a significant difference in the IRR of ADHD between the middle and the lowest tertile of the diet quality index, either in the univariate (IRR = 1.09, 95% CI: 0.81, 1.48) or multivariate models (IRR = 0.92, 95% CI: 0.63, 1.34), while the number of diagnoses of ADHD for the middle tertile in multivariate models was 8% lower than the lowest tertile of DQI-I. Students who played sports or PAs with a coach 1 to 3 times a week were less likely to use health care services for ADHD than students who never played sports or undertook PAs with a coach (IRR = 0.72, 95% CI: 0.52, 0.99). Use of a computer or playing video games greater than 5 h a day correlated to increased utilization of health care services for ADHD later in adolescence relative to less than one hour a day. From self-esteem categories, children who experienced more externalizing problems, low self-perception (e.g., body-esteem and hopes for the future) and low social-perception (e.g., being a bully-victim and lack of friends), had a higher number of physician visits for ADHD.

Stratified analyses by gender (Table 2) indicated that diet quality, PA, body weight, and externalization problems were associated with the number of diagnoses of ADHD in the same way in both genders. Boys residing in rural areas and in families with higher income ($20,001–$40,000/year) were less likely to contact health care providers for ADHD relative to those in urban areas and with lower household income (≤$20,000/year). Girls in families with higher parental educational levels and who spent more time watching TV had a lower likelihood of seeking health care services for health problems with ADHD relative to their peers with less parental education and less than one hour of TV viewing a day (Table 2). Self-perception and internalization problems among girls and social-perception and playing computer or video games among boys were also associated with higher service use for ADHD.

## 4. Discussion

We observed that poor diet quality, inadequate physical activities, and excessive use of computer and video games in childhood are associated with the increased use of health care services for ADHD during adolescence. These findings highlight the importance of intervention programs promoting active living and healthy eating among children for the prevention and reduction of service utilization of ADHD.

To our knowledge, this is the first population-based study among adolescents that reveals a prospective association between diet quality and clinical diagnoses of ADHD. Previous studies on the effect of dietary factors on ADHD have evaluated individual nutrients and some suggest that supplements of some foods or nutrients (e.g., omega-3 fatty acids, zinc, magnesium) and restriction or elimination of the detrimental foods (e.g., sugar) may help to reduce symptoms of ADHD among child and youth patients [49]. However, the findings from systematic review studies have been inconsistent regarding the effect of the individual diet factors [50,51]. It should be emphasized that individual food items and nutrients are often consumed in combinations that may cause a synergistic effect that influences the risk of ADHD [52].

Recent studies about the associations between dietary or nutrient patterns and ADHD in children and adolescents [24,25,26,27] have reported that unhealthy dietary patterns (e.g., western, fast-food, or junk food) are associated with an increased diagnosis of ADHD. We found that poor diet quality in childhood is associated with an elevated diagnosis of ADHD during adolescence. This is in line with the previous studies reporting that children and youth with unhealthy dietary patterns characterized as diet intake high in saturated and total fat, sugar, and salt, while low in whole grains, fish, fruits, and vegetables are more likely to experience ADHD symptoms [24,25,26,27,53]. We also observed that the association between diet quality and diagnosis of ADHD is stronger in girls than boys. The finding appears to be consistent with previous cross sectional studies [28]. A possible explanation for the gender difference is that girls may tend to make greater efforts than boys to eat healthy food, decreasing the energy intake in their diet in order to reduce their body weight. The girls with better diet quality are thus less likely to develop symptoms of ADHD than boys.

Our study adds to the existing research on the physical activity and ADHD relation by revealing a difference in the number of diagnoses for ADHD between children engaged in organized (e.g., with a coach) and unorganized (e.g., without a coach) sports. We found an independent association between PA with a coach, where children who participated in moderate physical activities (one to three times per week) were less likely to seek health care services for ADHD than children who never played sports or undertook PA with a coach. This is consistent with some previous studies showing that PA is inversely related to the diagnosis or symptoms of ADHD [21,31]. We also observed a significant relationship between physical activity without a coach and the diagnosis of ADHD. However, this association appeared to be not statistically significant when the effect of confounders was controlled. Previous studies indicate that PA has a positive effect on a number of mental health-related problems, such as improved self-esteem, emotional well-being, and peer-relationships [44,54]. The existing evidence also suggests that PA positively influences cognitive function and the academic performance of children and adolescents with or without ADHD [55,56,57]. These previous studies and our present results suggest that physical activity may be a protective factor for ADHD.

The present study revealed that children who spent excessive time (≥5 h/day) in playing computer and video games were more likely to be diagnosed with ADHD later than children who used the media less than one hour a day. This is consistent with studies showing that playing video games are linked to increased attention problems and ADHD among children and adolescents [32,34]. Evidence for the effect of TV viewing on ADHD is controversial [35,36]. We did not observe a relationship between watching TV and ADHD for boys, while girls who spent excessive time watching TV appeared to have less health problems with ADHD. The mechanism of the influence of TV watching on ADHD among youth needs further research. Exposure to some good TV programs such as educational programs may help alleviate ADHD among female adolescents [34].

The present study expands the analysis for associations between self-esteem in childhood and ADHD in adolescence. We found that children with more externalizing problems, low self-perception (body esteem and looks for future), and low social-perception (being victims of bullying and lack of friends) were more likely to receive a subsequent diagnosis of ADHD relative to children with high self-esteem on these components. These findings add to the existing literature on the relationship between self-esteem and mental health by addressing the impact of component-specific self-esteem in childhood on future ADHD [42].

We observed gender differences in the diagnosis of ADHD for the background variables of parental education, residency, household income, and the sedentary behaviours of the use of computer and video games and watching TV. These results underscore the importance of integrating gender differences in health promotion efforts to reduce the burden of the mental health disorder from ADHD among children and youth. Additionally, the gender differences in the association between the self-esteem variables and ADHD suggest that the influence of self-esteem of children on ADHD may be different among boys and girls. Poor social-perception is associated with increased diagnoses of ADHD among boys, while low self-esteem on self-perception and more internalizing problems are related to increased diagnoses of ADHD among girls.

In the present study, we found that physical activity, diet quality, and the use of a computer or playing video games in childhood had an impact on diagnoses of ADHD during adolescence. As ADHD is usually considered a chronic mental disorder with impairments persisting from childhood to adolescence and adulthood, identification of the onset of ADHD from childhood to early adulthood would be an additional interesting topic in future research. It may also be possible that the diet, physical activity, and sedentary behaviours of children and youth have also been affected by the symptoms of ADHD [28]. For example, children with ADHD may be more or less likely to attend organized physical activities [21]. Future research would warrant the elucidation of the reverse or bidirectional associations between ADHD and the health behaviours among children and youth using longitudinal data.

There are several strengths in this study. The analysis was conducted in a large representative sample of grade five students in the Canadian province of Nova Scotia with linked longitudinal administrative health data from childhood to early adulthood over an 8-year follow up period. The prospective design provides more robust evidence than cross-sectional studies for the directionality between the exposures and the mental health disorder outcome. The large sample allowed us to adjust the confounding influence of a variety of socio-demographic and self-esteem variables in the negative binomial regression analysis. The utilisation of health care provider diagnoses of mental health disorders provides valid and clinically meaningful assessments for ADHD.

Limitations should also be acknowledged when the study results are interpreted. The child physical activity, sedentary behaviours, and self-esteem were self-reported, subject to recall bias. Dietary assessment was also based on self-reporting which is prone to error, though the Harvard YAQ measure has been validated in this population of children [39]. The student participation rate of 51.1% is considered as fair to good for school based research. We mitigated the selection bias arising from non-responses by applying response weights. In addition, approximately 23% and 20% of students had missing information on household income and body weight. The adjustment for the confounding potential of household income and body weight may therefore be incomplete. Although we assessed health behaviors in childhood and ADHD throughout adolescence, reverse causation may still occur in cases where symptoms were present before the survey but their diagnosis occurred after the survey.

## 5. Conclusions

This study reveals that diet quality, physical activity, and the use of computer and video games among children are associated with increased diagnoses of attention deficit and hyperactivity behaviours later in adolescence. The findings suggest that broader investments in programs and interventions that promote healthy eating and active lifestyles in childhood may help prevent the development of ADHD and alleviate the public health burden and health care services associated with this mental disorder.

## Figures and Tables

**Table 1:** Socio-demographic characteristics of the grade five students and percentage (%) of receiving a diagnosis of ADHD, the Children’s Lifestyle and School Performance study, Nova Scotia, Canada.

Variable	% of Students, (*n* = 4875)	% of ADHDs, All Students	% of ADHDs, Boys (*n* = 2395)	% of ADHDs, Girls (*n* = 2480)
**DQI-I overall**				
Lowest tertile	33.6	9.37	14.39	4.59
Middle tertile	33.2	11.93	16.38	7.05
Highest tertile	33.2	7.74	12.36	3.73
**Physical activity without coach**				
Never	9.2	14.55	23.34	6.15
1 to 3 times/week	34.0	7.66	12.74	4.20
≥4 times/week	56.8	10.12	14.03	5.43
**Physical activity with coach**				
Never	37.5	11.77	18.57	5.98
1 to 3 times/week	44.8	7.77	11.74	3.95
≥4 times/week	17.8	10.28	13.59	6.04
**Using a computer or play video games**				
<1 h/day	42.7	7.63	12.30	4.65
1–2 h/day	38.7	8.94	12.84	4.70
3–4 h/day	12.3	11.00	13.83	6.49
≥5 h/day	6.3	25.46	29.22	11.77
**Watching TV**				
<1 h/day	14.6	11.07	16.34	6.93
1–2 h/day	40.2	8.03	12.12	4.47
3–4 h/day	29.2	8.86	13.12	4.71
≥5 h/day	16.0	13.79	19.57	4.98
**Gender**				
Boys	49.2	14.46	--	--
Girls	50.8	5.05	--	--
**Residence**				
Rural	32.7	8.16	11.88	4.36
Urban	67.3	10.42	15.77	5.38
**Parental education**				
Secondary education or less	28.28	10.72	16.14	6.12
College	34.89	10.66	16.07	5.37
University or above	29.60	7.81	11.05	4.21
Not reported	7.23	8.50	15.51	2.51
**Household income**				
≤$20,000	9.11	13.60	20.07	7.28
$20,001–$40,000	17.37	9.36	13.95	5.30
$40,001–$60,000	20.30	10.61	14.61	6.28
≥$60,001	29.99	7.89	11.35	4.44
Not reported	23.24	9.87	16.60	3.80
**Body weight**				
Normal weight	53.65	11.32	16.67	5.99
Overweight	18.40	9.11	13.70	4.82
Obese	7.80	8.76	12.93	4.05
Not assessed	20.15	6.18	9.52	3.26
**Self-esteem components**				
**Self-perception**				
High self-esteem	49.8	8.32	13.20	3.43
Low self-esteem	50.2	10.31	15.00	5.91
**Externalizing problems**				
High self-esteem	49.6	4.38	7.34	2.72
Low self-esteem	50.4	14.17	17.92	7.98
**Internalizing problems**				
High self-esteem	49.2	7.89	11.80	3.43
Low self-esteem	50.8	10.72	16.69	5.75
**Social-perception**				
High self-esteem	49.9	5.94	9.37	2.95
Low self-esteem	50.1	12.69	18.31	6.63

ADHD: attention deficit and hyperactivity disorder.

**Table 2:** Incidence rate ratios (IRRs) and 95% confidence intervals (CIs) for diagnoses of ADHD by the health behaviours, self-esteem, and socio-demographic variables among grade five students participating in the Children’s Lifestyle and School Performance study, Nova Scotia, Canada.

Variable	Unadjusted IRR (95% Cl)	Adjusted IRR (95% Cl)		
	All Students	All Students	Boys	Girls
**DQI-I overall**				
Lowest tertile	1.0	1.0	1.0	1.0
Middle tertile	1.09 (0.81, 1.48)	0.92 (0.63, 1.34)	0.94 (0.63, 1.40)	1.11 (0.60, 2.08)
Highest tertile	**0.60 (0.44, 0.84)**	**0.51 (0.35, 0.75)**	**0.63 (0.42, 0.93)**	**0.29 (0.15, 0.55)**
**Physical activity without coach**				
Never	1.0	1.0	1.0	1.0
1 to 3 times/week	**0.64 (0.43, 0.97)**	0.88 (0.51, 1.53)	0.76 (0.45, 1.29)	0.85 (0.32, 2.26)
≥4 times/week	0.72 (0.51, 1.02)	0.97 (0.58, 1.64)	0.77 (0.47, 1.26)	0.98 (0.37, 2.58)
**Physical activity with coach**				
Never	1.0	1.0	1.0	1.0
1 to 3 times/week	**0.67 (0.51, 0.89)**	**0.72 (0.52, 0.99)**	0.91 (0.63, 1.30)	**0.58 (0.33, 1.01)**
≥4 times/week	0.92 (0.64, 1.31)	0.86 (0.54, 1.36)	0.77 (0.48, 1.23)	1.01 (0.47, 2.20)
**Using a computer or playing video games**				
<1 h/day	1.0	1.0	1.0	1.0
1–2 h/day	**1.52 (1.12, 2.08)**	1.28 (0.86, 1.90)	1.11 (0.74, 1.67)	1.28 (0.67, 2.43)
3–4 h/day	**1.96 (1.30, 2.94)**	0.76 (0.47, 1.23)	0.79 (0.47, 1.33)	0.56 (0.24, 1.31)
≥5 h/day	**3.84 (2.67, 5.53)**	**2.67 (1.41, 5.05)**	**2.47 (1.41, 4.34)**	1.71 (0.50, 5.91)
**Watching TV**				
<1 h/day	1.0	1.0	1.0	1.0
1–2 h/day	0.86 (0.57, 1.29)	0.80 (0.51, 1.25)	0.84 (0.51, 1.38)	**0.46 (0.22, 0.93)**
3–4 h/day	1.13 (0.75, 1.70)	0.83 (0.51, 1.34)	0.93 (0.54, 1.58)	0.57 (0.22, 1.47)
≥5 h/day	1.54 (0.99, 2.40)	0.62 (0.34, 1.15)	1.03 (0.57, 1.85)	**0.15 (0.04, 0.57)**
**Gender**				
Boys	1.0	1.0	1.0	1.0
Girls	**0.31 (0.23, 0.42)**	**0.29 (0.21, 0.41)**	--	--
**Residence**				
Rural	1.0	1.0	1.0	1.0
Urban	**1.44 (1.09, 1.92)**	**1.54 (1.07, 2.20)**	**1.95 (1.37, 2.75)**	1.28 (0.63, 2.60)
**Parental education**				
Secondary education or less	1.0	1.0	1.0	1.0
College	0.95 (0.70, 1.29)	**0.63 (0.42, 0.94)**	1.16 (0.78, 1.73)	**0.32 (0.17, 0.59)**
University or above	**0.67 (0.48, 0.95)**	**0.63 (0.40, 0.98)**	1.08 (0.67, 1.74)	**0.37 (0.17, 0.80)**
**Household income**				
≤$20,000	1.0	1.0	1.0	1.0
$20,001–$40,000	0.61 (0.36, 1.05)	0.53 (0.25, 1.09)	**0.46 (0.23, 0.91)**	0.53 (0.20, 1.45)
$40,001–$60,000	0.82 (0.51, 1.31)	0.66 (0.33, 1.30)	0.65 (0.35, 1.22)	0.80 (0.29, 2.22)
≥$60,001	**0.56 (0.35, 0.90)**	0.72 (0.36, 1.42)	0.65 (0.35, 1.24)	0.77 (0.28, 2.11)
**Body weight**				
Normal weight	1.0	1.0	1.0	1.0
Overweight	0.80 (0.58, 1.11)	0.88 (0.59, 1.32)	0.88 (0.57, 1.37)	1.01 (0.54, 1.88)
Obese	0.66 (0.42, 1.05)	**0.49 (0.28, 0.86)**	**0.51 (0.29, 0.90)**	**0.33 (0.12, 0.92)**
**Self-esteem components**				
**Self-perception**				
High self-esteem	1.0	1.0	1.0	1.0
Low self-esteem	**1.32 (1.00, 1.73)**	**1.37 (1.00, 1.86)**	0.83 (0.59, 1.15)	**2.32 (1.36, 3.97)**
**Externalizing problems**				
High self-esteem	1.0	1.0	1.0	1.0
Low self-esteem	**3.81 (2.72, 5.32)**	**2.80 (2.01, 3.88)**	**3.04 (2.12, 4.37)**	**2.75 (1.58, 4.79)**
**Internalizing problems**				
High self-esteem	1.0	1.0	1.0	1.0
Low self-esteem	**1.43 (1.09, 1.88)**	1.28 (0.92, 1.77)	0.97 (0.68, 1.40)	**2.03 (1.12, 3.70)**
**Social-perception**				
High self-esteem	1.0	1.0	1.0	1.0
Low self-esteem	**2.63 (1.97, 3.53)**	**2.05 (1.44, 2.93)**	**2.21 (1.55, 3.17)**	1.49 (0.79, 2.79)

ADHD: attention deficit and hyperactive disorder. The model with adjusted IRR (95% Cl) values mutually adjusted for the variables in the table and energy intake. Estimates are weighted to represent grade five students in Nova Scotia. Bold values for IRRs and 95% CIs indicate statistical significance (*p* < 0.05).
